# Clinical Relevance of the LVEDD and LVESD Trajectories in HF Patients With LVEF < 35%

**DOI:** 10.3389/fmed.2022.846361

**Published:** 2022-05-13

**Authors:** Yu-Chen Chen, Shi-Chue Hsing, Yuan-Ping Chao, Yung-Wen Cheng, Chin-Sheng Lin, Chin Lin, Wen-Hui Fang

**Affiliations:** ^1^Department of Medical Education, Taipei Veterans General Hospital, Taipei, Taiwan; ^2^School of Medicine, National Defense Medical Center, Taipei, Taiwan; ^3^Department of Internal Medicine, Tri-Service General Hospital, National Defense Medical Center, Taipei, Taiwan; ^4^Division of Family Medicine, Department of Family and Community Medicine, Tri-Service General Hospital, Taipei, Taiwan; ^5^School of Medicine, National Defense Medical Center, Taipei, Taiwan; ^6^Division of Cardiology, Department of Internal Medicine, Tri-Service General Hospital, National Defense Medical Center, Taipei, Taiwan; ^7^Graduate Institute of Life Sciences, National Defense Medical Center, Taipei, Taiwan; ^8^School of Public Health, National Defense Medical Center, Taipei, Taiwan; ^9^Department of Family and Community Medicine, Tri-Service General Hospital, National Defense Medical Center, Taipei, Taiwan

**Keywords:** heart failure with reduced ejection fraction (HFrEF), ejection fraction, left ventricle end diastolic dimension (LVEDD), left ventricle end systolic dimension (LVESD), changes in ejection fraction

## Abstract

**Background:**

Certain variables reportedly are associated with a change in left ventricular ejection fraction (LVEF) in heart failure (HF) with reduced ejection fraction (HFrEF). However, literature describing the association between the recovery potential of LVEF and parameters of ventricular remodeling in echocardiography remains sparse.

**Methods:**

We recruited 2,148 HF patients with LVEF < 35%. All patients underwent at least two echocardiographic images. The study aimed to compare LVEF alterations and their association with patient characteristics and echocardiographic findings.

**Results:**

Patients with “recovery” of LVEF (follow-up LVEF ≥ 50%) were less likely to have prior myocardial infarction (MI), had a higher prevalence of atrial fibrillation (Af), were less likely to have diabetes and hypertension, and had a smaller left atrium (LA) diameter, left ventricular end-diastolic diameter (LVEDD) and left ventricular end-systolic diameter (LVESD), both in crude and in adjusted models (adjustment for age and sex). LVEDD cutoff values of 59.5 mm in men and 52.5 mm in women and LVESD cutoff values of 48.5 mm in men and 46.5 mm in women showed a year-to-year increase in the rate of recovery (follow-up LVEF ≥ 50%)/improvement (follow-up LVEF ≥ 35%), *p*-value < 0.05 in Kaplan–Meier estimates of the cumulative hazard curves.

**Conclusions:**

Our study shows that LVEDD and LVESD increments in echocardiography can be predictors of changes in LVEF in in HF patients with LVEF < 35%. They may be used to identify patients who require more aggressive therapeutic interventions.

## Introduction

Heart failure (HF) is a major public health problem associated with substantial morbidity, high mortality, and poor quality of life (1, 2). The new guidelines classify patients with HF into three categories according to left ventricular ejection fraction (LVEF): heart failure with preserved ejection fraction (HFpEF), heart failure with reduced ejection fraction (HFrEF), and heart failure with mid-range ejection fraction (HFmrEF). The diagnosis of HFrEF is defined by a reduced LVEF ≤ 40% (3). HFrEF patients have a significantly higher mortality rate than the other two types of HF patients (4, 5). With therapeutic advances over the past two decades, pharmacological therapy, coronary revascularization, and cardiac resynchronization therapy (CRT) have been used in the modern era. However, the absolute mortality among patients with HFrEF remains high and is comparable to that associated with other virulent diseases, such as cancer (6).

In a meta-analysis, improvement in LVEF and left ventricular (LV) volume was associated with lower rates of mortality among patients with HFrEF (7). Furthermore, a retrospective study reported that HF patients with recovered ejection fraction (defined as current LVEF > 40% but any previously documented LVEF < 40%) had lower mortality and fewer frequent hospitalizations than HFpEF and HFrEF patients (8). Therefore, in HFrEF, recovery of LV function is one of the treatment goals.

Shorter HF duration, lower baseline LVEF, non-ischemic cardiomyopathy, female sex, and no prior myocardial infarction (MI) were reportedly associated with an increase in LVEF in HFrEF patients (9, 10). However, echocardiographic findings other than LVEF were not included in previous studies. This study aimed to compare LVEF alterations and their association with patient characteristics and echocardiographic findings.

## Methods

### Study Design and Study Populations

This study was approved by the Institutional Review Board (TSGH-C202105049). It retrospectively and consecutively examined HF patients who were treated between April 2010 and September 2020 at a medical teaching hospital in northern Taiwan. Patients' clinical data were retrospectively reviewed without patients' written consent. Patients aged more than 18 years old with a primary or secondary diagnosis of HF at the time of the most recent office visit were included in the study. Participants were required to have an LVEF of <35% based on previous echocardiogram measurement and more than two echocardiographic images. That is, patients were required to have an echocardiogram showing LVEF < 35% as the baseline and one echocardiogram after that as the follow up image for analysis. Echocardiographic images were retrospectively collected in our hospital's database. The follow-up timing of echocardiography was the physicians' clinical decision. We were not involved in the decision making. We then excluded patients who had the following events or procedures between the baseline and follow-up assessment of LVEF: major cardiovascular surgery, cardiac device implantation, cancer, or heart transplant.

Recovery of LVEF was defined as an LVEF ≥ 50% on echocardiography follow-up, and improvement of LVEF was defined as an LVEF ≥ 35% but <50% on echocardiography follow-up in our analysis.

### Assessment of LVEF, Other Functional Parameters, and Variables

Echocardiographic findings, baseline demographics, clinical characteristics, comorbidities, and laboratory findings were obtained from patient charts and electronic health records by trained chart review specialists. Echocardiography was acquired by experienced cardiologists or technicians using standardized methods. LVEF was determined using the M-mode in the parasternal long-axis view or Simpson's biplane method. Parameters of ventricular and atrial remodeling, including changes in left atrium (LA) diameter, LV end-diastolic diameter (LVEDD), left ventricular end-systolic diameter (LVESD), interventricular septum (IVS), left ventricular posterior wall (LVPW), estimated pulmonary artery systolic pressure (PASP), and severity of valvular regurgitation from baseline, were also collected using the American Society of Echocardiography guidelines (11). LVEDD was measured at end-diastole on parasternal views. LVESD and LA diameter were measured from the parasternal long-axis view at end-systole. The thicknesses of IVS and LVPW were measured at end diastole. Continuous wave Doppler of the tricuspid regurgitation trace was used to measure and estimate PASP. We collected other laboratory data and HF medications from our electronic health records within seven days of the start of the study.

To further investigate the predictive performance of the recovery potential of LVEF and LVESD/LVEDD, Kaplan–Meier analysis was performed using follow-up data available in the echocardiography database for the chosen cutoff values, based on the Youden index (12). Time 0 was defined as the time of the patient's first echocardiography. The follow-up time continued to the time that event occurred (improvement/recovery) or was censored at the time of the patient's last echocardiography (if an event was not noted).

### Statistical Methods

Continuous variables of the general demographic data were expressed as the mean and standard deviation using Student's *t*-test. Categorical variables of the comorbidity described were analyzed using the χ^2^ or Fisher's exact test, as appropriate. A Cox proportional hazard model was used to estimate hazard ratios (HRs) and 95% confidence intervals (CIs) as measures of associations with LVEF improvement and LVEF recovery. The multivariable Cox proportional hazard model was used to adjust the potential confounding factors, and the adjusted variables were age and sex. A *p*-value < 0.05 was considered significant. Normality of distribution was tested by the Shapiro Wilk test in all continuous variables. If the distributions were skewed, we categorized these variables and reanalyzed their HRs. Statistical analyses were conducted using R software, version 3.4.4. In addition, we evaluated a Kaplan–Meier hazard curve to capture the hazard of the improvement of LVEF and recovery of LVEF, stratified by LVEDD and LVESD.

## Results

A total of 2,148 patients with baseline LVEF < 35% were included in this study. Among these patients, 516 (24%) had “no recovery” (follow-up LVEF < 35%), 714 (33.2%) had “improvement” (50% > follow-up LVEF ≥ 35%), and 918 (42.7%) patients had “recovery” (follow-up LVEF ≥ 50%). Compared to the other two groups of patients (Table 1), patients with “recovery” of LVEF were younger, were less likely to be male, had a higher blood pressure, were less likely to have previous MI and hyperlipidemia, had a higher prevalence of atrial fibrillation (Af), had a lower prevalence of diabetes and impaired kidney function, had a higher LVEF at baseline, had a smaller LVEDD and LVESD at baseline, had a larger IVS at baseline, and had a smaller aortic root diameter. Besides, HF medications at baseline was retrospectively collected and showed no significant difference.

**Table 1:** Characteristics among patients with different follow-up LVEF groups.

**Variable**	**“No recovery” (Follow-up LVEF <35%) (*n* = 516)**	**“Improvement” (50% > follow-up LVEF ≥35%) (*n* = 714)**	**“Recovery” (Follow-up LVEF ≥50%) (*n* = 918)**	***p-*value**
**Demography**				
Age (years)	67.07 ± 14.90	65.95 ± 14.22	63.25 ± 15.89	<0.001
Gender (male)	378 (73.3%)	539 (75.5%)	606 (66.0%)	<0.001
BMI (kg/m^2^)	24.65 ± 4.51	24.69 ± 4.09	24.50 ± 4.55	0.794
SBP (mmHg)	130.44 ± 24.88	141.10 ± 29.31	134.91 ± 29.77	0.013
DBP (mmHg)	82.76 ± 18.09	87.51 ± 20.52	84.85 ± 22.79	0.203
**Comorbidity**				
Hyperlipidemia	182 (35.3%)	280 (39.2%)	288 (31.4%)	0.004
COPD	94 (18.2%)	127 (17.8%)	181 (19.7%)	0.579
Prior myocardial infarction	71 (13.8%)	149 (20.9%)	96 (10.5%)	<0.001
Stroke	83 (16.1%)	130 (18.2%)	128 (13.9%)	0.064
Atrial fibrillation	57 (11.0%)	111 (15.5%)	163 (17.8%)	0.003
Diabetes mellitus	191 (37.0%)	292 (40.9%)	293 (31.9%)	0.001
Hypertension	290 (56.2%)	429 (60.1%)	506 (55.1%)	0.121
Chronic kidney disease	102 (19.8%)	161 (22.5%)	153 (16.7%)	0.011
**HF medications**				
Beta-blockers	261 (50.6%)	349 (48.9%)	477 (52.0%)	0.466
SGLT2 inhibitor	18 (3.5%)	19 (2.7%)	30 (3.3%)	0.672
ARNI	10 (1.9%)	24 (3.4%)	26 (2.8%)	0.326
ACEI/ARB	146 (28.3%)	224 (31.4%)	290 (31.6%)	0.388
MRA	77 (14.9%)	128 (17.9%)	168 (18.3%)	0.239
**Lab data**				
Creatinine (mg/dL)	2.28 ± 2.57	2.34 ± 2.69	2.13 ± 2.62	0.284
BUN (mg/dL)	36.65 ± 27.20	35.04 ± 25.87	32.82 ± 25.28	0.039
Fasting glucose (mg/dL)	128.86 ± 65.37	128.33 ± 61.86	129.69 ± 125.62	0.980
Na (mEq/L)	136.47 ± 5.15	137.26 ± 4.62	137.48 ± 4.69	0.002
K (mEq/L)	4.12 ± 0.64	4.07 ± 0.65	4.03 ± 0.71	0.082
Albumin (g/dL)	3.39 ± 0.55	3.40 ± 0.52	3.38 ± 0.54	0.818
WBC (K/μL)	9.33 ± 7.96	9.11 ± 4.35	9.41 ± 4.65	0.592
PLT (K/μL)	201.47 ± 82.02	203.61 ± 85.12	206.58 ± 84.48	0.572
Hemoglobin (g/dL)	12.25 ± 2.56	12.51 ± 2.66	12.46 ± 2.71	0.275
ALT (Unit/L)	83.98 ± 307.45	63.26 ± 216.79	83.17 ± 292.38	0.318
Triglyceride (mg/dL)	118.29 ± 74.24	126.01 ± 100.69	113.87 ± 65.45	0.021
Cholesterol (mg/dL)	149.86 ± 42.67	154.62 ± 45.74	151.14 ± 46.02	0.204
LDL (mg/dL)	94.52 ± 39.65	97.55 ± 39.42	95.36 ± 38.15	0.483
HDL (mg/dL)	38.85 ± 12.98	39.77 ± 12.15	41.11 ± 14.99	0.064
**Echocardiography**				
LVEDD (mm)	60.59 ± 8.97	57.14 ± 8.24	56.39 ± 8.92	<0.001
LVESD (mm)	50.10 ± 10.17	45.90 ± 9.12	45.43 ± 10.07	<0.001
IVS (mm)	11.46 ± 2.76	11.80 ± 2.55	11.50 ± 2.61	0.031
LVPW (mm)	9.88 ± 1.91	10.04 ± 2.09	9.91 ± 1.82	0.279
Left atrium diameter (mm)	45.20 ± 8.66	44.34 ± 8.97	44.00 ± 9.15	0.054
Aortic root diameter (mm)	34.17 ± 4.41	34.52 ± 4.44	33.86 ± 4.88	0.018
PASP (mmHg)	40.35 ± 14.99	38.36 ± 14.94	38.91 ± 14.72	0.064
LVEF (%)	25.95 ± 6.16	27.76 ± 5.62	27.12 ± 6.06	<0.001

*BMI, body mass index; SBP, systolic blood pressure; DBP, diastolic blood pressure; COPD, chronic obstructive pulmonary disease; SGLT2, sodium-glucose cotransporter 2; ARNI, angiotensin receptor-neprilysin inhibitor; ACEI, angiotensin-converting enzyme inhibitor; ARB, angiotensin receptor blocker; MRA, mineralocorticoid receptor antagonist; BUN, blood urea nitrogen; WBC, white blood cell; PLT, platelet count; ALT, alanine aminotransferase; LDL, low-density lipoprotein; HDL, high-density lipoprotein; LVEDD, left ventricular end-diastolic diameter; LVESD, left ventricular end-systolic diameter; IVS, interventricular septum; LVPW, left ventricular posterior wall; PASP, estimated pulmonary artery systolic pressure; LVEF, left ventricular ejection fraction*.

We checked normality in all continuous variables, and all the continuous variables were in skewed distributions. Therefore, we categorized these variables by normal values and reanalyzed their HRs in Tables 2, 3 as follows.

**Table 2:** The factors associated with improvement of LVEF (LVEF ≥ 35%) by Cox proportional hazard model.

**Variable**	**Crude HR (95% CI)**	***p-*value**	**Adjusted HR (95% CI)^#^**	***p-*value**
**Demography**				
BMI (kg/m^2^)		0.250		0.165
<18.5	1		1	
18.5–23.9	0.90 (0.68–1.20)	0.487	0.91 (0.69–1.22)	0.538
24–30	0.99 (0.74–1.32)	0.957	1.00 (0.75–1.34)	0.989
>30	0.81 (0.58–1.12)	0.201	0.78 (0.55–1.10)	0.162
SBP (mmHg)		0.110		0.062
<90	1		1	
90–120	0.66 (0.35–1.23)	0.191	0.62 (0.33–1.18)	0.144
121–140	0.58 (0.31–1.08)	0.085	0.54 (0.29–1.00)	0.052
>140	0.75 (0.41–1.39)	0.367	0.72 (0.39–1.33)	0.291
DBP (mmHg)		0.150		0.152
<60	1		1	
60–79	0.69 (0.47–1.02)	0.062	0.68 (0.46–1.00)	0.053
80–89	0.74 (0.49–1.12)	0.159	0.69 (0.46–1.05)	0.087
≥90	0.87 (0.60–1.28)	0.487	0.83 (0.57–1.21)	0.335
**Comorbidity**				
Hyperlipidemia	0.86 (0.78–0.95)	0.004	0.86 (0.78–0.96)	0.005
COPD	0.92 (0.81–1.04)	0.176	0.96 (0.84–1.09)	0.486
Prior myocardial infarction	1.09 (0.95–1.24)	0.235	1.10 (0.96–1.26)	0.179
Stroke	0.91 (0.80–1.04)	0.160	0.94 (0.82–1.07)	0.354
Atrial fibrillation	1.07 (0.94–1.21)	0.335	1.10 (0.96–1.25)	0.166
Diabetes mellitus	0.95 (0.86–1.05)	0.338	0.96 (0.86–1.06)	0.407
Hypertension	0.85 (0.77–0.94)	0.002	0.87 (0.79–0.96)	0.008
Chronic kidney disease	1.12 (0.99–1.27)	0.074	1.13 (1.00–1.28)	0.058
**Lab data**				
Creatinine (mg/dL)		0.079		0.024
≤ 1.2	1		1	
>1.2	1.09 (0.99–1.21)	0.079	1.12 (1.02–1.25)	0.024
BUN (mg/dL)		0.131		0.028
≤ 20	1		1	
>20	1.09 (0.98–1.21)	0.131	1.13 (1.01–1.26)	0.028
Fasting glucose (mg/dL)		0.121		0.103
60–99	1		1	
<60	0.78 (0.35–1.75)	0.548	0.79 (0.35–1.77)	0.565
100–126	1.00 (0.84–1.18)	0.956	0.99 (0.83–1.17)	0.901
>126	0.83 (0.69–0.99)	0.038	0.82 (0.68–0.98)	0.029
Na (mEq/L)		0.001		0.001
136–145	1		1	
<136	0.98 (0.62–1.57)	0.946	0.94 (0.59–1.51)	0.81
>145	1.55 (0.92–2.60)	0.1	1.53 (0.91–2.57)	0.109
K (mEq/L)		0.169		0.205
3.5–5.1	1		1	
<3.5	1.14 (0.99–1.32)	0.06	1.14 (0.99–1.31)	0.076
>5.1	1.04 (0.85–1.28)	0.683	1.04 (0.85–1.27)	0.712
Albumin (g/dL)		<0.001		<0.001
>3.5	1		1	
<2.5	1.65 (1.28–2.14)	<0.001	1.67 (1.29–2.17)	<0.001
2.5–3.5	1.20 (1.07–1.35)	0.002	1.25 (1.11–1.40)	<0.001
WBC (K/μL)		0.105		0.084
4–12	1		1	
<4	1.24 (0.96–1.60)	0.095	1.27 (0.99–1.64)	0.064
>12	1.10 (0.97–1.26)	0.139	1.10 (0.97–1.25)	0.153
PLT (K/μL)	1.00 (1.00–1.00)	0.531	1.00 (1.00–1.00)	0.627
Hemoglobin (g/dL)		0.001		<0.001
>10	1		1	
<8	1.47 (1.15–1.89)	0.002	1.49 (1.16–1.92)	0.002
8–10	1.20 (1.04–1.38)	0.014	1.22 (1.05–1.41)	0.008
ALT (Unit/L)		0.021		0.055
<40	1		1	
40–120	1.19 (1.03–1.36)	0.015	1.15 (1.00–1.32)	0.044
>120	1.18 (0.97–1.43)	0.102	1.17 (0.96–1.42)	0.112
Triglyceride (mg/dL)		0.142		0.050
≤ 200	1		1	
>200	0.87 (0.73–1.05)	0.142	0.83 (0.69–1.00)	0.050
Cholesterol (mg/dL)		0.906		0.613
<200	1		1	
≥200	1.01 (0.86–1.18)	0.906	0.96 (0.82–1.12)	0.613
LDL (mg/dL)		0.801		0.659
<100	1		1	
100–130	0.96 (0.83–1.11)	0.569	0.94 (0.81–1.08)	0.376
≥130	1.02 (0.87–1.19)	0.847	0.96 (0.82–1.13)	0.654
HDL (mg/dL)		0.403		0.534
≤ 50	1		1	
>50	0.93 (0.79–1.10)	0.403	0.95 (0.81–1.12)	0.534
**Echocardiography**				
LVEDD (mm)		<0.001		<0.001
≤ 53	1		1	
>53	0.73 (0.66–0.82)	<0.001	0.71 (0.64–0.79)	<0.001
LVESD (mm)		<0.001		<0.001
<40	1		1	
≥40	0.76 (0.68–0.85)	<0.001	0.73 (0.66–0.82)	<0.001
IVS (mm)		0.555		0.782
<11	1		1	
≥11	0.97 (0.88–1.07)	0.555	0.99 (0.89–1.09)	0.782
LVPW (mm)		0.259		0.213
<11	1		1	
≥11	0.93 (0.81–1.06)	0.259	0.92 (0.80–1.05)	0.213
Left atrium diameter (mm)		0.161		0.116
<40	1		1	
≥40	0.93 (0.84–1.03)	0.161	0.92 (0.83–1.02)	0.116
Aortic root diameter (mm)		0.935		0.759
<40	1		1	
≥40	0.99 (0.83–1.19)	0.935	1.03 (0.86–1.23)	0.759
PASP (mmHg)		0.440		0.317
<40	1		1	
≥40	1.04 (0.94–1.15)	0.440	1.05 (0.95–1.16)	0.317

# *All adjusted HR results were adjusted by sex and age*. *HR, hazard ratio; CI, confidence interval; BMI, body mass index; SBP, systolic blood pressure; DBP, diastolic blood pressure; COPD, chronic obstructive pulmonary disease; BUN, blood urea nitrogen; WBC, white blood cell; PLT, platelet count; AST, aspartate aminotransferase; ALT, alanine aminotransferase; LDL, low-density lipoprotein; HDL, high-density lipoprotein; LVEDD, left ventricular end-diastolic diameter; LVESD, left ventricular end-systolic diameter; IVS, interventricular septum; LVPW, left ventricular posterior wall; PASP, estimated pulmonary artery systolic pressure; LVEF, left ventricular ejection fraction*.

**Table 3:** The factors associated with recovery of LVEF (LVEF ≥ 50%) by Cox proportional hazard model.

**Variable**	**Crude HR (95% CI)**	***p-*value**	**Adjusted HR (95% CI)^#^**	***p-*value**
**Demography**				
BMI (kg/m^2^)		0.461		0.444
<18.5	1		1	
18.5–23.9	0.79 (0.55–1.13)	0.200	0.83 (0.58–1.19)	0.314
24–30	0.79 (0.55–1.13)	0.194	0.84 (0.58–1.22)	0.363
>30	0.71 (0.47–1.08)	0.111	0.70 (0.45–1.09)	0.112
SBP (mmHg)		0.047		0.034
<90	1		1	
90–120	0.51 (0.24–1.08)	0.079	0.51 (0.24–1.09)	0.082
121–140	0.38 (0.18–0.81)	0.012	0.37 (0.17–0.79)	0.010
>140	0.54 (0.26–1.11)	0.093	0.53 (0.26–1.11)	0.092
DBP (mmHg)		0.355		0.261
<60	1		1	
60–79	0.77 (0.48–1.24)	0.276	0.76 (0.47–1.23)	0.268
80–89	0.62 (0.36–1.07)	0.084	0.58 (0.33–1.00)	0.049
≥90	0.82 (0.51–1.32)	0.422	0.78 (0.48–1.25)	0.295
**Comorbidity**				
Hyperlipidemia	0.75 (0.65–0.86)	<0.001	0.74 (0.65–0.85)	<0.001
COPD	1.03 (0.87–1.21)	0.757	1.10 (0.93–1.30)	0.274
Prior myocardial infarction	0.65 (0.53–0.80)	<0.001	0.68 (0.55–0.84)	<0.001
Stroke	0.86 (0.71–1.03)	0.106	0.90 (0.74–1.08)	0.257
Atrial fibrillation	1.20 (1.02–1.43)	0.033	1.25 (1.05–1.49)	0.010
Diabetes mellitus	0.78 (0.68–0.89)	<0.001	0.78 (0.68–0.90)	0.001
Hypertension	0.82 (0.72–0.94)	0.004	0.85 (0.75–0.98)	0.021
Chronic kidney disease	0.99 (0.84–1.18)	0.947	0.99 (0.83–1.19)	0.953
**Lab data**				
Creatinine (mg/dL)		0.874		0.319
≤ 1.2	1		1	
>1.2	1.01 (0.88–1.16)	0.874	1.07 (0.93–1.23)	0.319
BUN (mg/dL)		0.354		0.073
≤ 20	1		1	
>20	1.07 (0.93–1.24)	0.354	1.14 (0.99–1.32)	0.073
Fasting glucose (mg/dL)		0.173		0.227
60–99	1		1	
<60	0.75 (0.24–2.37)	0.629	0.76 (0.24–2.39)	0.639
100–126	1.11 (0.88–1.40)	0.358	1.09 (0.87–1.38)	0.453
>126	0.86 (0.67–1.10)	0.229	0.85 (0.66–1.10)	0.222
Na (mEq/L)		0.092		0.063
136–145	1		1	
<136	0.91 (0.49–1.70)	0.767	0.86 (0.46–1.60)	0.626
>145	1.28 (0.64–2.56)	0.477	1.24 (0.62–2.46)	0.548
K (mEq/L)		0.109		0.145
3.5–5.1	1		1	
<3.5	1.21 (1.00–1.45)	0.046	1.19 (0.99–1.43)	0.067
>5.1	0.94 (0.71–1.24)	0.669	0.94 (0.71–1.23)	0.635
Albumin (g/dL)		0.001		0.001
>3.5	1		1	
<2.5	1.86 (1.36–2.55)	<0.001	1.78 (1.30–2.44)	<0.001
2.5–3.5	1.10 (0.95–1.29)	0.212	1.13 (0.97–1.32)	0.126
WBC (K/μL)		0.004		0.006
4–12	1		1	
<4	1.43 (1.03–1.98)	0.034	1.43 (1.03–1.98)	0.035
>12	1.27 (1.07–1.50)	0.005	1.25 (1.06–1.48)	0.008
PLT (K/μL)	1.00 (1.00–1.00)	0.307	1.00 (1.00–1.00)	0.763
Hemoglobin (g/dL)		0.001		0.001
>10	1		1	
<8	1.56 (1.11–2.19)	0.011	1.57 (1.11–2.20)	0.01
8–10	1.32 (1.09–1.60)	0.004	1.33 (1.10–1.60)	0.004
ALT (Unit/L)		0.048		0.126
<40	1		1	
40–120	1.23 (1.03–1.47)	0.022	1.17 (0.98–1.41)	0.083
>120	1.17 (0.91–1.51)	0.210	1.18 (0.92–1.52)	0.189
Triglyceride (mg/dL)		0.088		0.051
≤ 200	1		1	
>200	0.80 (0.63–1.03)	0.088	0.78 (0.60–1.00)	0.051
Cholesterol (mg/dL)		0.553		0.174
<200	1		1	
≥200	0.94 (0.76–1.16)	0.553	0.86 (0.70–1.07)	0.174
LDL (mg/dL)		0.440		0.199
<100	1		1	
100–130	0.93 (0.77–1.13)	0.455	0.93 (0.76–1.13)	0.444
≥130	0.87 (0.70–1.09)	0.234	0.82 (0.65–1.02)	0.078
HDL (mg/dL)		0.224		0.336
≤ 50	1		1	
>50	1.14 (0.92–1.41)	0.224	1.11 (0.90–1.37)	0.336
**Echocardiography**				
LVEDD (mm)		<0.001		<0.001
≤ 53	1		1	
>53	0.65 (0.57–0.75)	<0.001	0.65 (0.57–0.75)	<0.001
LVESD (mm)		<0.001		<0.001
<40	1		1	
≥40	0.69 (0.60–0.80)	<0.001	0.68 (0.59–0.78)	<0.001
IVS (mm)		0.174		0.553
<11	1		1	
≥11	0.91 (0.80–1.04)	0.174	0.96 (0.84–1.10)	0.553
LVPW (mm)		0.591		0.627
<11	1		1	
≥11	0.95 (0.80–1.14)	0.591	0.96 (0.80–1.14)	0.627
Left atrium diameter (mm)		0.028		0.042
<40	1		1	
≥40	0.86 (0.75–0.98)	0.028	0.87 (0.76–1.00)	0.042
Aortic root diameter (mm)		0.317		0.878
<40	1		1	
≥40	0.88 (0.69–1.13)	0.317	0.98 (0.76–1.26)	0.878
PASP (mmHg)		0.174		0.136
<40	1		1	
≥40	1.10 (0.96–1.25)	0.174	1.11 (0.97–1.27)	0.136

# *All adjusted HR results were adjusted by sex and age*. *HR, hazard ratio; CI, confidence interval; BMI, body mass index; SBP, systolic blood pressure; DBP, diastolic blood pressure; COPD, chronic obstructive pulmonary disease; BUN, blood urea nitrogen; WBC, white blood cell; PLT, platelet count; AST, aspartate aminotransferase; ALT, alanine aminotransferase; LDL, low-density lipoprotein; HDL, high-density lipoprotein; LVEDD, left ventricular end-diastolic diameter; LVESD, left ventricular end-systolic diameter; IVS, interventricular septum; LVPW, left ventricular posterior wall; PASP, estimated pulmonary artery systolic pressure; LVEF, left ventricular ejection fraction*.

The factors associated with LVEF improvement (LVEF ≥ 35%) are shown in Table 2. Subjects with LVEF improvement had both lower crude and adjusted models (adjustment for age and sex) of hyperlipidemia, hypertension, and baseline LVEDD/LVESD. Both higher crude and adjusted HRs of hypoalbuminemia, anemia, and liver function enzymes were also found. The adjusted HRs of creatinine and BUN were associated with LVEF improvement.

The factors associated with LVEF recovery (LVEF ≥ 50%) are shown in Table 3. Subjects with LVEF recovery had both lower crude and adjusted models (adjustment for age and sex) of hyperlipidemia, prior MI, diabetes mellitus, hypertension, baseline LVEDD/LVESD and LA diameter. Higher crude and adjusted HRs of Af, hypoalbuminemia, and anemia were also found. Liver function enzymes were associated with LVEF recovery in the crude mode, but its significance was lost after adjustment for age and sex. Since the inclusion of Af may introduce bias, we excluded Af patients and performed Supplementary Table S3. The results after excluding Af patients are similar.

**Figure 1:**
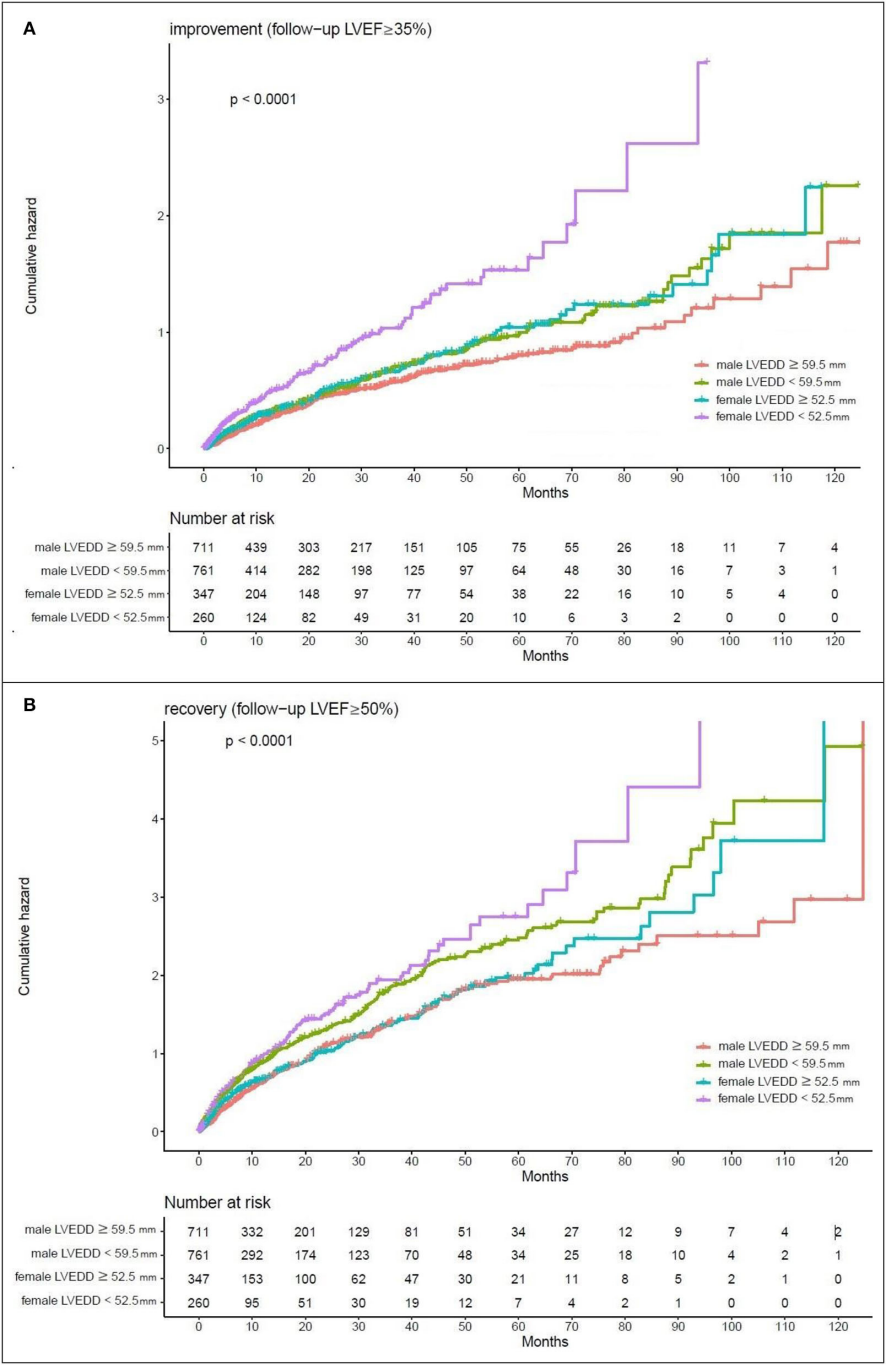
Kaplan-Meier estimates of the cumulative hazard for LVEDD cutoff values in LVEF alterations **(A)** LVEDD cutoff values of 59.5 mm in men and 52.5 mm in women showed a year-to-year increase in the rate of improvement (follow-up LVEF ≥ 35%). **(B)** LVEDD cutoff values of 59.5 mm in men and 52.5 mm in women showed a year-to-year increase in the rate of recovery (follow-up LVEF ≥ 50%).

**Figure 2:**
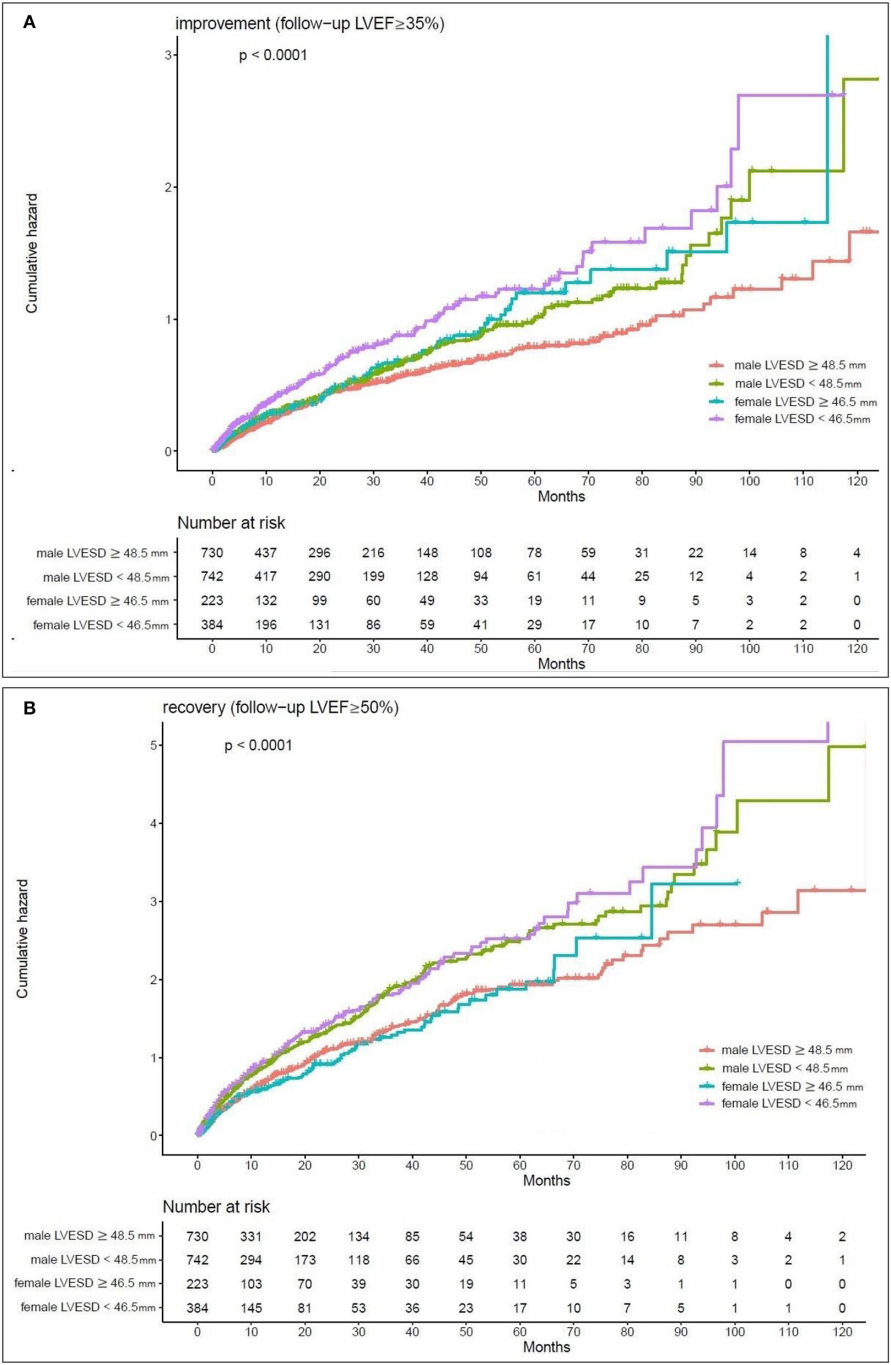
Kaplan-Meier estimates of the cumulative hazard for LVESD cutoff values in LVEF alterations **(A)** LVESD cutoff values of 48.5 mm in men and 46.5 mm in women showed a year-to-year increase in the rate of improvement (follow-up LVEF ≥ 35%). **(B)** LVESD cutoff values of 48.5 mm in men and 46.5 mm in women showed a year-to-year increase in the rate of recovery (follow-up LVEF ≥ 50%).

Kaplan–Meier estimates of the cumulative hazard curves for possible LVEF improvement (LVEF ≥ 35%) and recovery (LVEF ≥ 50%) during the follow-up period are depicted in Figures 1, 2. The median, mean, and interquartile range (IQR) of echocardiographic follow up of Kaplan–Meier analysis for LVEF improvement were 7.47, 15.19, and 17.5 months, respectively. The median, mean, and IQR of echocardiographic follow up of Kaplan-Meier analysis for LVEF recovery were 13.01, 21.96, and 18.66 months, respectively. LVEDD cutoff values of 59.5 mm in men and 52.5 mm in female; LVESD cutoff values of 48.5 mm in men and 46.5 mm in women were determined by the maximum of Youden index (12). In male patients with LVEDD < 59.5 mm and female patients with LVEDD < 52.5 mm, there was a year-to-year increase in the rate of improvement of LVEF and recovery of LVEF (*p*-value < 0.05). Male patients with LVESD < 48.5 mm and female patients with LVESD < 46.5 mm exhibited a higher hazard for improvement of LVEF and recovery of LVEF (*p-*value < 0.05).

## Discussion

The two major findings of the present study are as follows:

- Certain variables can be used to predict the response of change in LVEF. The data in our study showed that hyperlipidemia, prior MI, diabetes mellitus, hypertension, baseline LVEDD/LVESD and LA diameter were negatively associated with the recovery of LVEF. And Af, hypoalbuminemia, and anemia were positively associated with the recovery of LVEF.
- Both LVEDD and LVESD were associated with the recovery of LVEF. Furthermore, LVEDD cutoff values of 59.5 mm in men and 52.5 mm in women, and LVESD cutoff values of 48.5 mm in men and 46.5 mm in women predicted the improvement of LVEF and recovery of LVEF and can help physicians identify patients who are more likely to have a change in LVEF.

A correlation between the therapeutic effect of LVEF and mortality was reported in a meta-analysis. The regression analyses showed that a 5% increase in the mean EF change corresponded to a decreased odds ratio (OR) for mortality and favorable outcomes (7). Furthermore, one retrospective study reported that HF patients with recovered ejection fraction (defined as current LVEF > 40% but any previously documented LVEF < 40%) had lower mortality and fewer frequent hospitalizations than patients with HFpEF and HFrEF (8). Another prospective study showed that one in four treated patients showed recovery of LVEF, and patients with recovery of LVEF (defined as LVEF < 45% at baseline and ≥45% at 1 year) had better mortality and morbidity than patients with LVEF ≥45% and LVEF < 45% throughout follow-up (13). Another retrospective study reported that patients with previously reduced but now improved LVEFs to ≥50% had the highest overall quality of life score and lower dyspnea burden than those with HFpEF and HFrEF (14). Therefore, an increase in LV ejection function is one of the treatment goals in HFrEF and is associated with better outcomes. Pharmacological therapy, coronary revascularization, and cardiac resynchronization therapy are used to achieve this goal.

Certain variables reportedly predict the change in LVEF after treatment. However, the relationship between echocardiography features and the recovery potential of LVEF remains unclear. Shorter HF duration, lower baseline LVEF, non-ischemic cardiomyopathy or no prior myocardial infarction, and female sex were associated with LVEF improvement in previous studies (9, 10). However, one different point was found in our study. Baseline LVEF not associated with LVEF increment differed from the previous results that lower baseline LVEF was associated with LVEF improvement. Nevertheless, previous studies evaluating LV reverse remodeling used different assessments, such as LVEF increase 10% (9) or 5% (10), rather than a threshold >35 or 50%. The different settings might explain the differing results relating to the association between baseline LVEF and LVEF improvement.

To the best of our knowledge, this is the first study to examine the link between parameters of LV structure remodeling, such as LVEDD and LVESD, and LVEF increments in HFrEF patients. In addition, LVEDD cutoff values of 59.5 mm in men and 52.5 mm in women, and LVESD cutoff values of 48.5 mm in men and 46.5 mm in women may be useful and simple tools for the assessment of LVEF recovery potential.

In our study, we found prior MI, LA enlargement, dyslipidemia, diabetes, and hypertension are negatively association with recovery of LVEF.

The association of LA enlargement with HF has been well established. LA is correlated with LV function and change in LV filling pressure is associated with LA size (15). Thus, LA enlargement associated with various adverse cardiovascular events in previous literatures (16, 17). In one longitudinal study, LA enlargement was associated with severity of HF and predicted HFrEF (18). On the other hand, hypertension and diabetes are associated with an increased risk of developing HF and affect clinical outcomes (19). Hyperlipidemia is common in HF patients and is associated with worse prognosis.

Non-ischemic cardiomyopathy and no prior MI were more likely to increase LVEF in our study and previous reports. These results might be explained by different cardiac remodeling processes due to diverse pathological conditions. Necropsy studies have demonstrated that patients with congestive heart failure and significant coronary artery disease have gross myocardial scarring at autopsy, even in those without a clinical history of MI, angina, or Q waves (20), and scarring is uncommon in non-ischemic cardiomyopathy (21, 22). A cardiac magnetic resonance (CMR)-based study found scarring in 100% of patients with ischemic cardiomyopathy but in only 12% with non-ischemic cardiomyopathy, which is also consistent with necropsy studies (23). These findings explain why non-ischemic cardiomyopathy and no prior MI were more likely to increase LVEF in our study and previous reports. The recovery potential might differ due to different innate biological features and responses.

We also found patients with recovery of LVEF tend to have Af, hypoalbuminemia, and anemia.

An increasing prevalence of Af was observed with increasing EF (higher in HFpEF and HFmrEF compared to HFrEF) (24, 25). We found Af is association with recovery of LVEF. Because It's difficult to distinguish whether the HF presentation is acute or chronic HF, we could not directly link Af to acute HF deterioration. But catheter ablation, cardioversion, pharmacologic rhythm control, and rate control were all reportedly contributed to LVEF improvement in Af patient (26–29). The beneficial effect of Af in our study might relate to the combination of Af treatment.

Several studies demonstrated that there is an association between anemia, hypoalbuminemia and worse outcomes in HF patients regardless of LVEF, although it is unclear why these factors are associated with worse outcomes (30–33). Which is differed from our analysis that patients with recovery of LVEF tend to have hypoalbuminemia and anemia. But hypoalbuminemia and anemia may contribute to volume overload, acute exacerbation, or stress. Removal of excess of fluid, re-nutrition, and transfusion are easily to perform under current clinical condition and may improve LVEF.

An add-on strategy of drugs and devices is suggested in current HF treatment guidelines for HF patients with persistent low ejection fraction. The 2015 European Society of Cardiology (ESC) guidelines recommend ICD therapy in symptomatic patients with LVEF ≤ 35% after ≥3 months of optimal medical therapy. Despite the minor differences between their recommendations depending on the underlying heart disease, LVEF thresholds ≤ 35% were used to guide device-based therapy, including implantation of primary prevention ICDs and cardiac resynchronization therapy (CRT), both in American College of Cardiology (ACC) guidelines and ESC guidelines (34, 35). Therefore, we retrospectively enrolled HF patients with an LVEF < 35% for evaluation of the LVEF recovery potential in different underlying characteristics and baseline echocardiographic findings. In the current study, LV diameter results were associated with the recovery of LVEF. Furthermore, we also found that an LVEDD less than the cutoff level of 59.5 mm in men and 52.5 mm in women and LVESD less than the cutoff level of 48.5 mm in men and 46.5 mm in women independently predicted the improvement and recovery of LVEF. In addition, an increased LVEDD suggested a long and severe remodeling process of the LV, which was difficult to reverse. An echocardiography follow-up in hypertrophic cardiomyopathy found that increased LVEDD was associated with the HF endpoint (36). Another echocardiography follow-up in patients with recovered LVEF after medical treatment found that independent predictors of LVEF deterioration (from >45 to < 45%) included a high LVESD (37). Therefore, male patients with LVEDD increments larger than 59.5 mm and LVESD larger than 48.5 mm and female patients with LVEDD increments larger than 52.5 mm and LVESD larger than 46.5 mm may be a predictor of poor recovery potential of LVEF.

HF medications at baseline showed no significant difference in each groups in Table 1. An increase in LVEF was not observed in the randomized clinical trials with angiotensin receptor-neprilysin inhibitor (ARNI) and sodium-glucose cotransporter 2 (SGLT2) inhibitors (38, 39). Nevertheless, prior randomized clinical trials described improvements in LVEF with beta-blockers, angiotensin-converting enzyme inhibitor (ACEI), angiotensin receptor blocker (ARB), mineralocorticoid receptor antagonist (MRA) therapy (13). Because HF medications at baseline was retrospectively collected from our hospital's medical record, patients who labeled as no HF medications might receive treatment in other hospital. On the other hands, dosage of HF medications was not analyzed. Clinical guidelines recommend slowly up titrating to maximal tolerated doses (19). So, patients might receive medications at variable dosing. And patients may receive follow-up and start treatment a period of time after echocardiography in outpatient department. Above factors could lead to the result that we did not find an association between HF medications at baseline and improvement in LVEF.

Our study drew its strength from a large sample size, a longitudinal design rather than a cross-sectional design, and a well-validated echocardiographic finding.

However, our analysis has some limitations that need to be acknowledged. First, all patients were from a center in Taiwan using a longitudinal design. Sampling bias and selection bias are deemed inevitable. We could not assure the generalizability of the results to other populations and could not establish a cause–effect relationship. Second, the duration and etiology of HF, which are reportedly associated with LVEF improvement in previous literature, were not mentioned in our study. Although it is unclear why the duration of heart failure modifies recovery potential, it may be related to scar burden and the cumulative level of myocyte injury (39). Third, the schedule of follow-up echocardiography was clinically driven and thus highly variable. The frequency and timing of follow-up echocardiography may be related to other heart failure outcomes. Patients who died before the follow-up echocardiography were recorded as lost to follow-up in our database, so the effect on left ventricular function improvement might be overestimated. Another potential issue is the definition of recovery. We chose to include patients with LVEF from ≤ 35% at baseline to >50% based on LVEF thresholds used to guide the cardiac device implantation of primary prevention ICDs. Whereas, this finding is congruent with prior studies, other cutoffs used to define improved LVEF include >40% [40], >45% (37), and >50% (20). A fixed improvement % of LVEF was also used, such as an LVEF increase of 10% (9) or 5% (10). Standardization of definitions in LVEF improvement/recovery is likely needed for prospective studies. Fourth, concomitant disease of Af was included, but treatment for Af was not mentioned in our analysis. Catheter ablation, pharmacologic conversion, or rate control treatment (e.g., digoxin) may have an impact on LVEF and the remodeling process. In addition, the LVEF of Af disease is variable from beat to beat. Finally, an add-on strategy of pharmacological therapy and devices, including ACEI, beta-blockers, MRA, and ARNI, is suggested in current HF treatment guidelines. However, doses of HF medications at baseline and HF medications at follow-up were not analyzed in the study. Patients might receive variable medications, at variable dosing and for variable durations or may not receive appropriate HF treatment. All the above factors may limit the generalizability of the findings.

## Conclusions

In our study, no previous MI history, no hyperlipidemia, no hypertension, non-diabetic, smaller baseline LVEDD/LVESD and LA diameter were associated with 50% or greater LVEF recovery. LVEDD cutoff values of 59.5 mm in men and 52.5 mm in women and LVESD cutoff values of 48.5 mm in men and 46.5 mm in women were associated with LVEF increments. These data may inform discussions on therapies for HFrEF and can be the bases for future research on parameters of ventricular remodeling.

## Data Availability Statement

The original contributions presented in the study are included in the article/Supplementary Material, further inquiries can be directed to the corresponding author.

## Ethics Statement

The studies involving human participants were reviewed and approved by Tri-Service General Hospital. Written informed consent for participation was not required for this study in accordance with the national legislation and the institutional requirements.

## Author Contributions

Y-CC contributed to drafting of the manuscript and critical revision of the manuscript. S-CH contributed to algorithm development, drafting of the manuscript, and submission process. Y-PC contributed to data acquisition, concept generation, and the methodology. Y-WC contributed to data acquisition and concept generation. C-SL contributed to concept generation and the methodology. CL contributed to the methodology, data acquisition, and interpretation. W-HF contributed to concept generation, the methodology, data acquisition and interpretation, and approval of the article authors. All authors contributed to the article and approved the submitted version.

## Funding

This study was funded by the Ministry of Science and Technology, Taiwan (110-2314-B-016-008 to W-HF), the Tri-Service General Hospital, Taiwan (TSGH-E-111217 to W-HF), and National Defense Medical Center (MND-MAB-D-111143 to W-HF).

## Conflict of Interest

The authors declare that the research was conducted in the absence of any commercial or financial relationships that could be construed as a potential conflict of interest.

## Publisher's Note

All claims expressed in this article are solely those of the authors and do not necessarily represent those of their affiliated organizations, or those of the publisher, the editors and the reviewers. Any product that may be evaluated in this article, or claim that may be made by its manufacturer, is not guaranteed or endorsed by the publisher.
