# Supplementary material for: Clinical Relevance of the LVEDD and LVESD Trajectories in HF Patients With LVEF < 35%
Source: Front Med (Lausanne). 2022 May 13;9:846361. doi: 10.3389/fmed.2022.846361 (PMC9136034; doi:10.3389/fmed.2022.846361)
Supplement: Supplementary file 1 [file Table_1.docx]

**Table S3 | The factors associated with recovery of LVEF (LVEF ≥50%), excluded patients who having atrial fibrillation, by Cox proportional hazard model.**

| **Variable** | **Crude HR (95% CI)** | ***p value*** | **Adjusted HR (95% CI)#** | ***p value*** |
| --- | --- | --- | --- | --- |
| **Demography** |  |  |  |  |
| BMI (kg/m2) |  | 0.374 |  | 0.313 |
| <18.5 | 1 |  | 1 |  |
| 18.5-23.9 | 0.84 (0.57 - 1.22) | 0.349 | 0.86 (0.59 - 1.26) | 0.454 |
| 24-30 | 0.80 (0.55 - 1.17) | 0.254 | 0.85 (0.57 - 1.25) | 0.399 |
| >30 | 0.68 (0.43 - 1.06) | 0.089 | 0.66 (0.41 - 1.05) | 0.081 |
| SBP (mmHg) |  | 0.110 |  | 0.054 |
| <90 | 1 |  | 1 |  |
| 90-120 | 0.48 (0.19 - 1.22) | 0.122 | 0.44 (0.17 - 1.13) | 0.087 |
| 121-140 | 0.36 (0.14 - 0.91) | 0.031 | 0.32 (0.12 - 0.81) | 0.016 |
| >140 | 0.49 (0.20 - 1.22) | 0.128 | 0.46 (0.18 - 1.14) | 0.093 |
| DBP (mmHg) |  | 0.404 |  | 0.215 |
| < 60 | 1 |  | 1 |  |
| 60-79 | 0.82 (0.50 - 1.37) | 0.457 | 0.80 (0.48 - 1.34) | 0.403 |
| 80-89 | 0.60 (0.33 - 1.11) | 0.105 | 0.53 (0.29 - 0.98) | 0.042 |
| ≥ 90 | 0.85 (0.51 - 1.42) | 0.538 | 0.78 (0.47 - 1.30) | 0.340 |
| **Comorbidity** |  |  |  |  |
| Hyperlipidemia | 0.75 (0.64 - 0.88) | <0.001 | 0.74 (0.64 - 0.87) | <0.001 |
| COPD | 1.05 (0.87 - 1.26) | 0.642 | 1.10 (0.91 - 1.33) | 0.323 |
| Prior myocardial infarction | 0.64 (0.51 - 0.80) | <0.001 | 0.66 (0.53 - 0.83) | <0.001 |
| Stroke | 0.86 (0.69 6- 1.07) | 0.175 | 0.89 (0.71 - 1.11) | 0.282 |
| Diabetes mellitus | 0.80 (0.68 - 0.93) | 0.004 | 0.80 (0.68 - 0.93) | 0.004 |
| Hypertension | 0.85 (0.74 - 0.98) | 0.027 | 0.87 (0.76 - 1.01) | 0.070 |
| Chronic kidney disease | 1.02 (0.84 - 1.23) | 0.839 | 1.01 (0.84 - 1.22) | 0.918 |
| **Lab data** |  |  |  |  |
| Creatinine (mg/dL) |  | 0.458 |  | 0.141 |
| ≤ 1.2 | 1 |  | 1 |  |
| > 1.2 | 1.06 (0.91 - 1.23) | 0.458 | 1.12 (0.96 - 1.30) | 0.141 |
| BUN (mg/dL) |  | 0.173 |  | 0.036 |
| ≤ 20 | 1 |  | 1 |  |
| > 20 | 1.12 (0.95 - 1.31) | 0.173 | 1.19 (1.01 - 1.40) | 0.036 |
| Fasting glucose (mg/dL) |  | 0.311 |  | 0.323 |
| 60-99 | 1 |  | 1 |  |
| < 60 | 1.22 (0.89 - 1.67) | 0.217 | 1.22 (0.89 - 1.67) | 0.225 |
| 100-126 | 1.00 (0.74 - 1.37) | 0.985 | 1.01 (0.74 - 1.38) | 0.929 |
| > 126 | 0.91 (0.68 - 1.21) | 0.515 | 0.91 (0.68 - 1.21) | 0.513 |
| Na (mEq/L) |  | 0.293 |  | 0.240 |
| 136-145 | 1 |  | 1 |  |
| < 136 | 0.99 (0.84 - 1.17) | 0.906 | 1.00 (0.84 - 1.18) | 0.958 |
| > 145 | 1.31 (0.93 - 1.85) | 0.127 | 1.34 (0.95 - 1.89) | 0.097 |
| K (mEq/L) |  | 0.006 |  | 0.013 |
| 3.5-5.1 | 1 |  | 1 |  |
| < 3.5 | 1.38 (1.13 - 1.68) | 0.001 | 1.34 (1.10 - 1.64) | 0.003 |
| > 5.1 | 1.05 (0.79 - 1.40) | 0.752 | 1.03 (0.77 - 1.37) | 0.843 |
| Albumin (g/dL) |  | 0.001 |  | 0.001 |
| > 3.5 | 1 |  | 1 |  |
| < 2.5 | 1.92 (1.38 - 2.67) | <0.001 | 1.86 (1.34 - 2.59) | <0.001 |
| 2.5-3.5 | 1.07 (0.90 - 1.27) | 0.453 | 1.09 (0.92 - 1.30) | 0.302 |
| WBC (K/μL) |  | 0.002 |  | 0.002 |
| 4-12 | 1 |  | 1 |  |
| < 4 | 1.43 (1.00 - 2.03) | 0.049 | 1.42 (1.00 - 2.03) | 0.051 |
| > 12 | 1.34 (1.12 - 1.60) | 0.001 | 1.33 (1.11 - 1.59) | 0.002 |
| PLT (K/μL) | 1.00 (1.00 - 1.00) | 0.322 | 1.00 (1.00 - 1.00) | 0.846 |
| Hemoglobin (g/dL) |  | 0.004 |  | 0.003 |
| > 10 | 1 |  | 1 |  |
| < 8 | 1.25 (0.85 - 1.84) | 0.250 | 1.26 (0.86 - 1.85) | 0.241 |
| 8-10 | 0.79 (0.64 - 0.97) | 0.022 | 0.78 (0.63 - 0.96) | 0.017 |
| ALT (Unit/L) |  | 0.193 |  | 0.252 |
| < 40 | 1 |  | 1 |  |
| 40-120 | 1.15 (0.94 - 1.41) | 0.169 | 1.10 (0.90 - 1.35) | 0.339 |
| >120 | 1.21 (0.92 - 1.58) | 0.171 | 1.23 (0.94 - 1.60) | 0.138 |
| Triglyceride (mg/dL) |  | 0.116 |  | 0.075 |
| ≤ 200 | 1 |  | 1 |  |
| > 200 | 0.81 (0.62 - 1.05) | 0.116 | 0.79 (0.61 - 1.02) | 0.075 |
| Cholesterol (mg/dL) |  | 0.416 |  | 0.142 |
| < 200 | 1 |  | 1 |  |
| ≥ 200 | 0.91 (0.73 - 1.14) | 0.416 | 0.84 (0.67 - 1.06) | 0.142 |
| LDL (mg/dL) |  | 0.393 |  | 0.195 |
| < 100 | 1 |  | 1 |  |
| 100-130 | 0.91 (0.74 - 1.13) | 0.392 | 0.92 (0.74 - 1.13) | 0.421 |
| ≥130 | 0.86 (0.67 - 1.09) | 0.214 | 0.80 (0.63 - 1.02) | 0.077 |
| HDL (mg/dL) |  | 0.114 |  | 0.178 |
| ≤ 50 | 1 |  | 1 |  |
| > 50 | 1.21 (0.96 - 1.53) | 0.114 | 1.18 (0.93 - 1.49) | 0.178 |
| **Echocardiography** |  |  |  |  |
| LVEDD (mm) |  | <0.001 |  | <0.001 |
| ≤ 53 | 1 |  | 1 |  |
| > 53 | 0.68 (0.59 - 0.80) | <0.001 | 0.69 (0.59 - 0.81) | <0.001 |
| LVESD (mm) |  | <0.001 |  | <0.001 |
| < 40 | 1 |  | 1 |  |
| ≥ 40 | 0.67 (0.57 - 0.79) | <0.001 | 0.66 (0.56 - 0.78) | <0.001 |
| IVS (mm) |  | 0.743 |  | 0.730 |
| < 11 | 1 |  | 1 |  |
| ≥ 11 | 0.98 (0.84 - 1.13) | 0.743 | 1.03 (0.88 - 1.19) | 0.730 |
| LVPW (mm) |  | 0.896 |  | 0.871 |
| < 11 | 1 |  | 1 |  |
| ≥ 11 | 1.01 (0.84 - 1.23) | 0.896 | 1.02 (0.84 - 1.23) | 0.871 |
| Left atrium diameter (mm) |  | 0.029 |  | 0.043 |
| < 40 | 1 |  | 1 |  |
| ≥ 40 | 0.85 (0.73 - 0.98) | 0.029 | 0.86 (0.74 - 1.00) | 0.043 |
| Aortic root diameter (mm) |  | 0.060 |  | 0.253 |
| < 40 | 1 |  | 1 |  |
| ≥ 40 | 0.75 (0.56 - 1.01) | 0.060 | 0.84 (0.62 - 1.13) | 0.253 |
| PASP (mmHg) |  | 0.139 |  | 0.137 |
| < 40 | 1 |  | 1 |  |
| ≥ 40 | 1.12 (0.96 - 1.30) | 0.139 | 1.12 (0.96 - 1.30) | 0.137 |

#All adjusted HR results were adjusted by sex and age.

HR: hazard ratio; CI: confidence interval; BMI: body mass index; SBP: systolic blood pressure; DBP: diastolic blood pressure; COPD: chronic obstructive pulmonary disease; BUN: blood urea nitrogen; WBC: white blood cell; PLT: platelet count; AST: aspartate aminotransferase; ALT: alanine aminotransferase; LDL: low-density lipoprotein; HDL: high-density lipoprotein; LVEDD: left ventricular end-diastolic diameter; LVESD: left ventricular end-systolic diameter; IVS: interventricular septum; LVPW: left ventricular posterior wall; PASP: estimated pulmonary artery systolic pressure; LVEF: left ventricular ejection fraction
