# Supplementary material for: Clinical Relevance of the LVEDD and LVESD Trajectories in HF Patients With LVEF < 35%
Source: Front Med (Lausanne). 2022 May 13;9:846361. doi: 10.3389/fmed.2022.846361 (PMC9136034; doi:10.3389/fmed.2022.846361)
Supplement: Supplementary file 2 [file Data_Sheet_1.DOCX]

LVEDD - male

| value | sensitivity | 1-specificity | Yuden Index |
| --- | --- | --- | --- |
| 59.5 | 0.624 | 0.435 | 0.189 |
| 58.5 | 0.669 | 0.483 | 0.186 |
| 57.5 | 0.718 | 0.533 | 0.185 |
| 56.5 | 0.758 | 0.593 | 0.165 |
| 55.5 | 0.809 | 0.649 | 0.16 |

LVEDD - female

| value | sensitivity | 1-specificity | Yuden Index |
| --- | --- | --- | --- |
| 52.5 | 0.684 | 0.539 | 0.145 |
| 56.5 | 0.441 | 0.301 | 0.140 |
| 51.5 | 0.721 | 0.586 | 0.135 |
| 59.5 | 0.324 | 0.191 | 0.133 |
| 58.5 | 0.36 | 0.229 | 0.131 |

LVESD - male

| value | sensitivity | 1-specificity | Yuden Index |
| --- | --- | --- | --- |
| 48.5 | 0.629 | 0.451 | 0.178 |
| 47.5 | 0.661 | 0.49 | 0.171 |
| 49.5 | 0.581 | 0.411 | 0.170 |
| 50.5 | 0.53 | 0.365 | 0.165 |
| 44.5 | 0.772 | 0.609 | 0.163 |

LVESSD - female

| value | sensitivity | 1-specificity | Yuden Index |
| --- | --- | --- | --- |
| 46.5 | 0.544 | 0.316 | 0.228 |
| 45.5 | 0.566 | 0.361 | 0.205 |
| 47.5 | 0.478 | 0.287 | 0.191 |
| 44.5 | 0.603 | 0.416 | 0.187 |
| 48.5 | 0.426 | 0.248 | 0.178 |
